# Sources of Stress and Their Associations With Mental Disorders Among College Students: Results of the World Health Organization World Mental Health Surveys International College Student Initiative

**DOI:** 10.3389/fpsyg.2020.01759

**Published:** 2020-07-30

**Authors:** Eirini Karyotaki, Pim Cuijpers, Yesica Albor, Jordi Alonso, Randy P. Auerbach, Jason Bantjes, Ronny Bruffaerts, David D. Ebert, Penelope Hasking, Glenn Kiekens, Sue Lee, Margaret McLafferty, Arthur Mak, Philippe Mortier, Nancy A. Sampson, Dan J. Stein, Gemma Vilagut, Ronald C. Kessler

**Affiliations:** ^1^Department of Global Health and Social Medicine, Harvard Medical School, Boston, MA, United States; ^2^Department of Clinical, Neuro and Developmental Psychology, Vrije Universiteit Amsterdam, Amsterdam, Netherlands; ^3^Department of Epidemiologic and Psychosocial Research, National Institute of Psychiatry Ramón de la Fuente Muñiz, Mexico City, Mexico; ^4^Health Services Research Unit, IMIM Hospital del Mar Medical Research Institute, Barcelona, Spain; ^5^CIBER en Epidemiología y Salud Pública, Madrid, Spain; ^6^Pompeu Fabra University, Barcelona, Spain; ^7^Department of Psychiatry, Columbia University, New York, NY, United States; ^8^Department of Psychology, Stellenbosch University, Stellenbosch, South Africa; ^9^Universitair Psychiatrisch Centrum – Katholieke Universiteit Leuven, Leuven, Belgium; ^10^Department of Clinical Psychology and Psychotherapy, Friedrich–Alexander University Erlangen–Nürnberg, Erlangen, Germany; ^11^School of Psychology, Curtin University, Perth, WA, Australia; ^12^Center for Public Health Psychiatry, Katholieke Universiteit, Leuven, Belgium; ^13^Department of Health Care Policy, Harvard Medical School, Boston, MA, United States; ^14^School of Biomedical Sciences, Ulster University, Londonderry, United Kingdom; ^15^Department of Psychiatry, The Chinese University of Hong Kong, Shatin, Hong Kong; ^16^Department of Psychiatry and Mental Health and South African Medical Council Research Unit on Risk and Resilience in Mental Disorders, University of Cape Town and Groote Schuur Hospital, Cape Town, South Africa

**Keywords:** stress, college students, mental disorder, anxiety disorders, mood disorders, substance use disorders

## Abstract

The college years are stressful for many students. Identifying the sources of stress and their relative importance in leading to clinically significant emotional problems may assist in the development of targeted stress management interventions. The current report examines the distribution and associations of perceived stress across major life areas with 12-month prevalence of common mental disorders in a cross-national sample of first-year college students. The 20,842 respondents were from 24 universities in 9 countries that participated in the World Health Organization World Mental Health International College Student Initiative. Logistic regression analysis examined associations of current perceived stress in six life areas (financial situation, health, love life, relationships with family, relationships at work/school, problems experienced by loved ones) with six types of 12-month mental disorders (major depressive disorder, bipolar disorder, generalized anxiety disorder, panic disorder, alcohol use disorder, drug use disorder). Population attributable risk proportions (PARPs) were calculated to estimate the upper-bound potential effects of interventions focused on perceived stress in reducing prevalence of mental disorders. The majority of students (93.7%) reported at least some stress in at least one of the six areas. A significant dose-response association was found between extent of stress in each life area and increased odds of at least one of the six disorders. The multivariable models that included all stress measures were significant for all disorders (*F* = 20.6–70.6, *p* < 0.001). Interpretation of PARPs as representing causal effects of stresses on disorders suggests that up to 46.9–80.0% of 12-month disorder prevalence might be eliminated if stress prevention interventions were developed to block the associations of stress with these disorders.

## Introduction

The college years are an important developmental period when young people transition from adolescence to young adulthood (Arnett, 2000; Baghurst and Kelley, 2014). College students, especially first-year students, face numerous challenges in making successful transitions. These challenges can be exacerbated by life stresses that vary across students depending on objective circumstances and psychological resilience (Heffer and Willoughby, 2017). Although some level of stress is a normal part of life and can even be motivating in certain contexts (Robotham and Julian, 2006) high levels of stress pose a threat to well-being (Cohen et al., 2019) and academic attainment (Eicher et al., 2014). Targeted stress management interventions can be developed (Stillwell et al., 2017; Feiss et al., 2019) but no large-scale epidemiological studies have investigated the relative magnitudes of these stresses among college students across a range of life areas. Nor have studies estimated the potential importance of stress either overall or within individual life areas in accounting for common college student mental disorders.

The current report presents data on the prevalence and severity of stresses reported by first-year college students across nine countries in mental health needs assessment surveys carried out by the World Health Organization (WHO) World Mental Health International College Student Initiative (WMH-ICS). WMH-ICS implements such surveys among college students around the world (Alonso et al., 2019; Auerbach et al., 2019; Cuijpers et al., 2019) and implements, evaluates, and disseminates scalable preventive and ameliorative interventions guided by information obtained in the surveys about important areas of unmet need for treatment (Harrer et al., 2018; Harrer et al., 2019; Karyotaki et al., 2019). One area of particular interest is the development of psychotherapies that prioritize stressors most relevant to students (Cuijpers et al., 2018). The objective of the present paper was to investigate what the most common sources of stress are among college students and to what extent this may account for 12-month mental disorders that are common among college students (Auerbach et al., 2018).

## Materials and Methods

### Study Design

Results are based on WMH-ICS surveys carried out with first-year students in a convenience sample of 24 colleges and universities (8 private and 16 public; henceforth referred to as colleges) in nine countries (Australia, Belgium, Germany, Hong Kong, Mexico, Northern Ireland, South Africa, Spain, and the United States) between September 2014 and February 2018 (Supplementary Tables 1, 2). The surveys used web-based self-report questionnaires and an attempt was made to assess all first-year students in each college. A total of 21,369 questionnaires were completed, with sample sizes ranging from 208 in Hong Kong to 8,076 in Mexico. The weighted (by achieved sample size) mean response rate across surveys was 45.6%. The current sample includes five additional colleges and one additional country (a survey in Hong Kong) compared to earlier WMH-ICS survey reports (Alonso et al., 2018, 2019; Auerbach et al., 2018, 2019; Mortier et al., 2018b; Bruffaerts et al., 2019; Ebert et al., 2019). Poststratification weights (Groves and Couper, 2012) were used to adjust for non-response bias based on socio-demographic information provided by officials from the participating schools.

**TABLE 1:** Twelve-month prevalence estimates of DSM-IV disorders in the WMH-ICS sample (*n* = 20,842)^1^.

	**%**	**(*SE*)**
**(I) Mood**		
Major depressive disorder	13.4	(0.3)
Bipolar disorder	3.3	(0.1)
**(II) Anxiety**		
Generalized anxiety disorder	13.1	(0.3)
Panic disorder	3.8	(0.2)
**(III) Substance use**		
Alcohol use disorder	6.4	(0.2)
Drug use disorder	1.9	(0.1)

*WMH-ICS, World Mental Health Surveys International College Student Initiative; SE, MI-adjusted standard error. ^1^Prevalence estimates are based on weighted data. See the text for a discussion of weighting.*

**TABLE 2:** Distribution of responses to the perceived stress questions in the WMH-ICS sample (*n* = 20,842)^1^.

	**None**		**Mild**		**Moderate**		**Severe**		**Very**	
	**%**	**(*SE*)**	**%**	**(*SE*)**	**%**	**(*SE*)**	**%**	**(*SE*)**	**%**	**(*SE*)**
Financial situation	31.4	(0.4)	29.0	(0.4)	24.6	(0.4)	10.6	(0.3)	4.5	(0.2)
Own health	35.7	(0.4)	32.4	(0.4)	22.1	(0.3)	7.6	(0.2)	2.3	(0.1)
Love life	33.2	(0.4)	27.5	(0.4)	22.8	(0.3)	11.3	(0.3)	5.2	(0.2)
Relationships with family	43.3	(0.4)	25.5	(0.3)	18.5	(0.3)	8.4	(0.2)	4.2	(0.2)
Relationships at school/work	47.1	(0.4)	28.3	(0.4)	16.9	(0.3)	5.9	(0.2)	1.9	(0.1)
Problems of loved ones^2^	25.2	(0.3)	27.4	(0.4)	25.9	(0.3)	14.6	(0.3)	6.9	(0.2)

*WMH-ICS, World Mental Health Surveys International College Student Initiative; SE, MI-adjusted standard error. ^1^Distributions are based on weighted data. See the text for a discussion of weighting. ^2^Combined across two separate questions. See text for details.*

The analyses reported here are based on 20,842 respondents (Supplementary Tables 1, 2). The remaining 527 respondents were excluded either because they reported not being either male or female (*n* = 79), reported not being a full-time student (*n* = 413), or were missing information on gender or fulltime versus part-time student status (*n* = 39). Students that did not identify as male or female were excluded because they are the subject of more focused analyses that will be described in a future report. Part-time students were excluded because they were quite different from full-time students on many characteristics important to the analysis, including having an older age distribution and being more likely to be married, employed full-time, and have children. Students with missing gender data were excluded because gender is a core variable in many WMH-ICS analyses.

### Data Collection Procedures

Data collection for this study was conducted using an online self-report survey that was distributed to all incoming first year students, who were invited to participate in various settings. This included during registration, health screenings or via a separate email invitation. The main strategies used to address the issue of low participation rates among students, were repeated rounds of contact together with an offer of small tokens in exchange for completing the survey.

After an initial contact, non-responders received follow-up personalized emails and ten colleges provided low-cost incentives (e.g., movie passes, a raffle for store coupons), while one survey team used an additional strategy of providing incentives to a random sample of non-responders. In Mexico, students were enrolled in the survey at a number of mandatory events (e.g., student health evaluations and tutoring sessions) where time was allocated specifically for completing the survey.

At all survey sites, the local ethics or institutional review committee reviewed and approved the protocol to ensure protection of human subjects, in line with appropriate international and local guidelines. All students were requested to sign an informed consent prior to participation. Participation was voluntarily. Detailed information on the organizations responsible for ethics approval for each survey is available at this link: http://www.hcp.med.harvard.edu/wmh/ftpdir/IRB_EthicsApproval_WMH-ICS.pdf. On completion of the survey, all students were provided with information on how to access mental health services at their institution, and additional in-depth information on services was provided to any student who reported recent and/or severe suicidal thoughts and behaviors (Mortier et al., 2018a).

### Measures

#### Current Perceived Stress in Different Life Areas

The MIDUS self-report scale of perceived stress was used to assess stresses currently (at the time of survey) experienced by respondents in a series of seven life domains relevant to college students: financial situation, own health, love life, relationships with family, relationships with people at work/school, health and wellbeing of loved ones, and other problems experience by loved ones (Kessler et al., 2004). We did not ask about the stresses associated with academic performance, as the survey was designed to be administered at the beginning of the academic year, which would be before such stresses became manifest. The scale asked respondents to rate “how much stress you have in each the following areas of life” for each of the life domains, with response options of none, mild, moderate, severe, and very severe coded 0–4 for purpose of the current analyses. This scale has demonstrated good internal consistency (α = 0.83) in a sample of college students (Kiekens et al., 2016). Responses to the two questions about problems experienced by loved ones were combined into one category due to the high correlation between the two responses (Pearson *r* = 0.72). This was done by assigning the higher of the two scores to the composite, which is henceforth referred to as “problems of loved ones.”

#### Mental Disorders

The WMH-ICS survey instrument was developed to assess six common mental disorders: major depressive disorder (MDD), bipolar I-II disorder (BPD), generalized anxiety disorder (GAD), panic disorder (PD), attention deficit/hyperactivity disorder (ADHD), and drug abuse or dependence (DUD; referring to “drug use disorder”), using the Composite International Diagnostic Interview Screening Scales (CIDI-SC; Kessler et al., 2013a, b).

The CIDI-SC scales are short validated self-report screening scales designed to assess 12-month prevalence of disorders based on the definitions and criteria of the Diagnostic and Statistical Manual of Mental Disorders, Fourth Edition (DSM-IV). The CIDI-SC scales have been shown to have good concordance with blinded clinical diagnoses [Area Under the Curve (AUC) of 0.70–0.78] (Kessler et al., 2013a, b; Ballester et al., 2019). The CIDI-SC has shown good concordance for all diagnoses in a college student sample (AUCs > 0.8). A seventh disorder, alcohol abuse or dependence (AUD; referring to “alcohol use disorder”), was assessed with the Alcohol Use Disorders Identification Test (AUDIT; Saunders et al., 1993). Alcohol use disorder is defined as either a total score of ≥16 or a score of 8–15 with ≥4 on the AUDIT dependence questions (Babor et al., 2001). The AUDIT has been shown to have good concordance with clinical diagnoses (AUC of 0.78–0.91) (Reinert and Allen, 2002).

#### Socio-Demographics and College-Related Factors

We controlled for several socio-demographic variables in the analyses reported here: gender (male, female), age (16–18, 19, 20+), parental education (higher of two parents using the categories secondary school or less [low], some postsecondary education [medium], college graduate or more [high]), parental marital status (currently married to each other versus either divorced or at least one deceased), religion (Christian, other religion, no religion), and sexual orientation (heterosexual, gay or lesbian, bisexual, asexual, not sure, other). The survey included further questions on sexual attraction to and sexual contact with males and females and the responses yielded four distinct categories: heterosexual with no same-sex attraction, heterosexual with some same-sex attraction, non-heterosexual without same-sex sexual contact, and non-heterosexual with same-sex sexual contact. We also controlled for self-reported high school academic ranking (categorized as top 50% or bottom 50%) and country where the survey was conducted.

### Analysis Methods

#### Weighting and Imputation

Data were weighted within each college to adjust for discrepancies between population socio-demographic distributions provided by college administrators and sample distributions. Standard post-stratification methods were used for weighting (Groves and Couper, 2012). Comparison of the distributions between respondents and populations found only one consistent difference prior to weighting: that females had a higher response rate than males. This was adjusted for in the weighting. As mentioned above, Spain used an “end-game” strategy to increase recruitment. Non-respondents at the end of the normal recruitment period were more aggressively followed-up. The “hard to reach” respondents who were eventually interviewed were assigned a weight equal to 1/p, where p represented the proportion of non-respondents at the end of the normal recruitment period that was included in the end game, to adjust for the under-sampling of these hard-to-recruit respondents. An equal sum of weights was given to each country in the analysis.

Multiple imputation (MI) by chained equations (Buuren, 2012) was used to adjust for item-missing data, random internal sampling of survey sections in some countries to reduce survey length, and missing data due to skip logic errors that occurred in a few countries. Twenty MI replicate datasets were used. All reported standard errors (SEs) and degrees of freedom were adjusted using Rubin’s rules for combining multiple imputed estimates (Rubin, 2004).

#### Estimating Prevalence

The prevalence estimates presented here report on 12 month disorders, based on weighted within college proportions. The corresponding SEs are estimated using Rubin’s rules to account for the imputation of missing data.

#### Estimating Associations

Logistic regression models were used to estimate the associations of perceived stress with 12-month disorders, controlling for socio-demographics (country, gender, age, sexual orientation, religion, parental education, marital status of parents, and self-reported high school ranking). Both univariable models with only one stress measure and multivariable models with all stress measures were estimated to evaluate predictive associations of the stress measures with the disorders. A logistic link function was used in all the models. We began by estimating linear associations of scores on the 0–4 stress scales with the outcomes and then tested for a difference in the slope discriminating between respondents with no stress (scores of 0) versus mild stress (scores of 1) compared to transitions across higher pairs of scores on the stress scale. This was done both because 0 was by far the most common score on each scale and because our intuition was that the difference between any stress and no stress might be more important (i.e., have a higher slope) than differences across the remainder of the range. This test was carried out using standard spline regression coding methods (Marsh and Cormier, 2002). The multivariable models that combined information about stresses across life areas were then estimated using the predictors in the best-fitting univariable models. We evaluated the combined predictive effects of groups of parameters using MI-adjusted Wald *F* tests.

#### Carrying Out Intervention Simulations

Population attributable risk proportions (PARPs; Greenland and Drescher, 1993) were calculated for the best-fitting multivariable model for each disorder in order to estimate the potential effects of interventions that either reduced stress or reduced the effects of stress on mental disorders. PARPs can be interpreted in this way based on the provisional assumptions that the stresses assessed are causal risk factors for mental disorders and that the causal effects of these stresses are captured by the logistic regression coefficients.

Simulations were used to calculate the PARPs. This began by estimating the expected prevalence of each mental disorder based on the best-fitting prediction model for that disorder. Expected prevalence estimates were then recalculated seven different times: six of these fixed the logit (or logits) of the predictor(s) in one area of stress to 0. The seventh set the logits for the predictors across all stress areas to 0. PARP was defined as the ratio of (i) the difference in the predicted prevalence of a given mental disorder in the observed data versus if the logits were set to 0 (ii) divided by the predicted prevalence of the disorder in the observed data. SEs of the PARP estimates were generated using the Jackknife Repeated Replication simulation method, where each college was treated as a stratum and two random half-samples per college were generated and treated as sampling error calculation units, with the whole Jackknife Repeated Replication estimation process embedded within the MI replicate design (Rust and Rao, 1996). All analyses were carried out using SAS Version 9.4 (SAS Institute Inc, 2014).

## Results

### Socio-Demographic Distribution of the Sample

Most respondents were 16–18 years old (57.6%), female (54.7%), heterosexual with no same-sex attraction (76.2%), and Christian (70.6%) (Supplementary Table 3). Most respondents reported having a parent with a high level of education (55.9%) and that their parents were both living and still married to each other (75.4%).

### Distributions of Mental Disorders and Stress

Twelve-month prevalence of mental disorders ranged from a high of 13.4% for MDD to a low of 1.9% for DUD (Table 1). Between two-thirds and three-fourths of respondents experienced at least mild stress about problems experienced by loved ones (74.8%), their own financial situation (68.6%), love life (66.8%), and their health (64.3%) (Table 2). About half of respondents experienced at least mild stress about their relationships with family (56.7%) and with people at school/work (52.9%). Among respondents who reported any stress in the given life area, mean (SE) severity was 2.0 (0.01) for problems of loved ones, 1.9 (0.01) for their own financial situation and love life, 1.8 (0.01) for relationships with family, 1.7 (0.01) for own health, and 1.6 (0.01) for relationships with people at school/work.

Aggregated across life areas, 93.7% of respondents had at least one type of stress rated at least mild (Table 3). The smallest proportion of students had stress in only one of the six life areas (8.1%) and the proportions were successively larger for students who had stress in between 2 (11.8%) and 6 (26.9%) areas. 73.8% of students had stress in at least three life areas. There was a significant positive association between number of stresses experienced and mean stress severity within the areas in which stress was experienced (Pearson *r* = 0.36, *p* < 0.001), with the mean (SE) stress level across stress areas where stress was experienced ranging from a low of 1.4 (0.02) among people that experienced stress in only 1 life area to a high of 2.0 (0.01) among people that experienced stress in all six areas.

**TABLE 3:** Distribution and association between number of areas of perceived stress and mean stress severity in the WMH-ICS sample (*n* = 20,842)^1^.

	**%**	**(*SE*)**	**Mean^2^**	**(*SE*)**	**(*n*)^3^**
**Number of areas**					
1	8.1	(0.2)	1.4	(0.0)	(1,685)
2	11.8	(0.3)	1.5	(0.0)	(2,435)
3	13.8	(0.3)	1.6	(0.0)	(2,886)
4	15.6	(0.3)	1.7	(0.0)	(3,270)
5	17.5	(0.3)	1.8	(0.0)	(3,665)
6	26.9	(0.4)	2.0	(0.0)	(5,579)
1 or more	93.7	(0.0)	1.7	(0.0)	(19,519)

*WMH-ICS, World Mental Health Surveys International College Student Initiative; SE, MI-adjusted standard errors. ^1^Number of areas with at least mild stress. Statistics are based on weighted data. See the text for a discussion of weighting. ^2^Means are calculated only for the areas of stress reported as present. ^3^Sample sizes are unweighted.*

### Univariable Associations of Stress With Mental Disorders

Univariable models were estimated for stress in one area at a time predicting each type of mental disorder controlling for the socio-demographic variables. The associations of socio-demographics with mental disorders are not reported here, as they have been reported previously (Auerbach et al., 2018). A significant positive association was found between each of the 6 0–4 linear stress measures and each of the six mental disorders, with the 36 odds ratios (ORs) in the range 1.3–1.7 (Supplementary Tables 4–9).

### Multivariable Associations of Stress With Mental Disorders

Multivariable models documented powerful associations of the stress measures with odds of all mental disorders (*F* = 44.3–213.3, *p* < 0.001) (Table 4). We also considered the possibility that interactions involving number of types of stress were important predictors, but none of these was significant in exploratory analyses. Stresses involving financial situation and love life were significant in predicting all six disorders. Stresses involving relationships with family members were significant in predicting four disorders. Stresses involving relationships at work/school were significant in predicting three disorders. Stresses involving the student’s own health were significant in predicting two disorders. And stresses involving problems of loved ones were significant in predicting one disorder. GAD was predicted by all six types of stress, MDD and BPD by 4, PD and DUD by 3, and AUD by 2 types of stress.

**TABLE 4:** Multivariable associations of perceived stress with 12-month presence of DSM-IV mental disorders in the WMH-ICS sample (*n* = 20,842).

	**Major depressive disorder**		**Bipolar disorder**		**Generalized anxiety disorder**		**Panic disorder**		**Alcohol use disorder**		**Drug use disorder**	
	**OR**	**(95% CI)**	**OR**	**(95% CI)**	**OR**	**(95% CI)**	**OR**	**(95% CI)**	**OR**	**(95% CI)**	**OR**	**(95% CI)**
**Financial situation**												
Linear	1.1*	(1.1-1.2)	1.3*	(1.2-1.4)			1.2*	(1.1-1.3)	1.2*	(1.1-1.3)	1.2*	(1.1-1.4)
Low spline					1.3*	(1.2-1.3)						
**Own health**												
Linear					1.2*	(1.1-1.2)	1.5*	(1.4-1.6)				
**Love life**												
Linear			1.2*	(1.1-1.3)					1.3*	(1.3-1.4)		
Low spline	1.5*	(1.4-1.6)			1.3*	(1.2-1.4)	1.2*	(1.1-1.3)			1.4*	(1.2-1.5)
**Relationships with family**												
Any	1.4*	(1.2-1.6)	1.7*	(1.3-2.2)								
Linear					1.1*	(1.0-1.1)					1.2*	(1.1-1.4)
Low spline	1.1*	(1.0-1.2)	1.2*	(1.1-1.4)								
**Relationships at work/school**												
Linear	1.2*	(1.1-1.3)	1.2*	(1.1-1.3)	1.3*	(1.2-1.3)						
**Problems of loved ones**												
Low spline					1.2*	(1.1-1.3)						
**All stresses F**	187.3*		84.8*		213.3*		71.1*		114.9*		44.3*	

WMH-ICS, World Mental Health International College Student Initiative; OR, odds ratio; CI, confidence interval. *Significant at the 0.05-level, using MI-adjusted tests. ^1^All models based on weighted data controlling for the socio-demographic variables in Supplementary Table 3. Any = 0–1 dichotomy coded 0 for respondents who reported no stress in the life area and coded 1 for respondents who reported mild, moderate, severe, or very severe stress in the life area; Linear = 0–4 variable for respondents who reported no stress (0), mild (1), moderate (2), severe (3), or very severe (4) stress in the life area; Low spline = 1–4 variable for respondents who reported no stress (1), mild (1), moderate (2), severe (3), or very severe (4) stress in the life area.

### Population Attributable Risk Proportions

The PARP estimates suggest that the stresses considered here are associated with 46.9–80.0% of the prevalence of the disorders considered in this sample. This means that the projected prevalence estimates of the disorders among respondents with none of the stresses were only between 20% (i.e., 100% – 80.0%) and 53.1% (i.e., 100% – 46.9%) as high as the observed prevalence estimates (Table 5). Decomposition showed that stress due to love life was the strongest correlate of MDD (23.8%) and AUD (32.2%), stress in relationships at work/school was the strongest correlate of GAD (20.3%), stress in relationships with family was the strongest correlate of BPD (43.9%), stress due to one’s own health was the strongest correlate of PD (42.6%), and financial stress was the strongest correlate of DUD (29.1%). The component PARPs sum to more than the overall PARP because of correlations among the types of stress.

**TABLE 5:** Population attributable proportions of 12-month DSM-IV disorders associated with perceived stress based on the multivariable models in the WMH-ICS sample (*n* = 20,842)^1^.

	**Major depressive disorder**		**Bipolar disorder**		**Generalized anxiety disorder**		**Panic disorder**		**Alcohol use disorder**		**Drug use disorder**	
	**Est**	**(*SE*)**	**Est**	**(*SE*)**	**Est**	**(*SE*)**	**Est**	**(*SE*)**	**Est**	**(*SE*)**	**Est**	**(*SE*)**
Financial situation	14.6	(4.1)	40.2	(6.3)	14.1	(2.8)	28.0	(15.5)	22.4	(5.4)	29.1	(11.0)
Own health	–		–		16.4	(4.1)	42.6	(13.5)	–		–	
Love life	23.8	(2.5)	30.7	(10.1)	17.1	(2.2)	11.5	(8.9)	32.2	(5.0)	23.9	(7.6)
Relationships with family	21.3	(5.0)	43.9	(10.7)	9.8	(4.6)	–		–		25.1	(11.4)
Relationships at work/school	16.3	(3.2)	23.2	(7.5)	20.3	(3.2)	–		–		–	
Problems of loved ones	–		–		14.0	(2.9)	–		–		–	
All stresses	57.7	(2.7)	80.0	(3.7)	64.2	(1.7)	62.0	(9.9)	46.9	(4.2)	57.6	(8.5)

*WMH-ICS, World Mental Health International College Student Initiative; SE, standard error. ^1^Based on the models in Table 4. Standard errors were calculated using the Jackknife Repeated Replications simulation method embedded within MI replicates.*

## Discussion

Previous surveys of college students have consistently implicated finances, health, love life, relationships with friends, and relationships with family as sources of stress during the college years (Darling et al., 2007; Lust et al., 2008; Stallman, 2010; Beiter et al., 2015). We built on these previous surveys in developing the brief WMH-ICS assessment of perceived stress. Our results are compelling in documenting strong associations of perceived stress across all the life areas considered with the full range of common mental disorders previously found to be associated with serious role impairments among college students (Auerbach et al., 2016).

Among the strengths of the study are the large cross-national sample, the investigation of stress across a number of different life areas, and the evaluation of all the mental disorders found in previous cross-national research to be most strongly related to academic role performance among college students (Mahmoud et al., 2012; Ibrahim et al., 2013). There are also several notable limitations. First, the colleges were a convenience sample, limiting the external validity of results. Second, the survey response rates were low across most participating sites. This is typical of other large-scale college student surveys (Eisenberg et al., 2013; Paul et al., 2015). Previous research has found a weak association between response rates and non-response bias (Groves, 2006). In the case of surveys about mental disorders this bias might be in the direction of overestimating the prevalence of mental disorders (Mortier et al., 2018a). As a result, the low response rates might have led to an over-estimation of disorder prevalence and might also have introduced some bias into the estimate of the associations between perceived stress and mental disorders.

A third limitation is that the stress measures assessed perceptions of stress rather than objective stressor characteristics. More extensive self-report questionnaires exist to assess objective stressor characteristics. Semi-structured interviews can also be used to obtain more objective measures of chronic stressors (Harkness and Monroe, 2016). Both of the latter approaches are labor intensive and burdensome to respondents, though, leading us to consider them infeasible for WMH-ICS surveys, as the latter were designed to be brief screening surveys that assessed a wide range of risk factors and disorders. However, given the very high PARPs found in the current report, it might make sense to carry out more focused surveys of objective chronic stressors in parallel with future WMH-ICS surveys.

A fourth limitation is that it is impossible to draw clear causal interpretations of the associations between stress and mental disorders using a cross-sectional non-experimental design of the sort used in the WMH-ICS surveys. The reason for this is that complex reciprocal associations are known to exist between chronic stress and mental disorders (Kassel et al., 2007; Liu and Alloy, 2010; Newman et al., 2013; Majd et al., 2017). It is noteworthy in this regard that a prior WMH-ICS report showed that 83% of the active mental disorders among first-year college students in our surveys were reported to have started in childhood or adolescence; that is, well *before* matriculation to college (Auerbach et al., 2016). This raises the possibility that the stresses associated with mental disorders among first-year college students might be chronic stressors that began prior to matriculation or acute stresses that were consequences of pre-existing mental disorders rather than causal risk factors for 12-month disorder onset-persistence. Finally, in the present work we focused on general stress sources in different meaningful areas of life. Building upon these findings, future studies should incorporate specific stressors such as adjustment to the new environment.

Within the context of these limitations, we found that nearly all students experience stress in at least one of the life areas considered here; that the great majority of students experience at least some stress in multiple life areas; that perceived stress in each of these areas is an important correlate of at least one of the disorders considered here; that the joint predictive associations of these multiple stresses are additive; and that these joint predictive associations account for substantial proportions (46.9–80.0%) of the mood, anxiety, and substance use disorders considered here.

Although our data do not permit us to ascertain the causal role of stress in leading to these strong associations with the mental disorders considered here, experimental studies show that stress management interventions effectively reduce perceived stress, mood, and anxiety problems through a combination of problem-focused and appraisal-focused strategies (Van der Klink et al., 2001; Regehr et al., 2013; Conley et al., 2015; Breedvelt et al., 2018). Based on this evidence, the interventions needed to address the range of stresses experienced by college students would presumably include both training in problem-focused coping strategies to reduce the occurrence and severity of objective stressors and in emotion-focused coping strategies to reduce the impact of unmodifiable stressors (Baqutayan, 2015; Smith et al., 2016; Breedvelt et al., 2019; Stanisławski, 2019). Although the most consistently significant stresses found in the WMH-ICS data are in the areas of finances and love life, interventions would need to help students develop strategies to manage a wide range of stresses given that each type of stress considered in our analysis was associated with increased odds of at least 1 type of common mental disorder. Furthermore, the great majority of students with mental disorders reported stresses in several domains, so training in the management of stress overload might be of value.

Although it is as yet unclear how effective specific types of scalable therapies focused on the stresses of first-year college students would be in reducing mental disorders and improving academic performance, colleges and universities could play an important role in maximizing the potential value of such interventions by participating in systematic efforts to implement and evaluate pragmatic trials of the sort being developed by WMH-ICS to reduce the stresses of college students. To the extent that these interventions alleviate stress, they might also reduce prevalence of mental disorders, and, by virtue of the strong associations known to exist between mental disorders and academic performance (Bruffaerts et al., 2018; Wilks et al., 2020) improve the academic performance and subsequent life-long well-being of students.

## Who WMH-ICS Collaborators

Australia: Penelope Hasking (PI), Mark Boyes, David Preece (School of Psychology, Curtin University); Belgium: Ronny Bruffaerts (PI), Erik Bootsma, Koen Demyttenaere, Glenn Kiekens (KU Leuven); France: Mathilde Husky (PI), Université de Bordeaux; Viviane Kovess-Masfety, Ecole des Hautes Etudes en Santé Publique; Germany: David D. Ebert (PI), Matthias Berking, Marvin Franke, Fanny Kählke (Friedrich-Alexander University Erlangen Nuremberg); Harald Baumeister, Ann-Marie Küchler (University of Ulm); Hong Kong: Arthur Mak (PI), Jenny Ho, Idy Chou (The Chinese University of Hong Kong); Siu Oi-ling (Lingnan University); Mexico: Corina Benjet (PI), Yesica Albor, Guilherme Borges, María Elena Medina-Mora (Instituto Nacional de Psiquiatría Ramón de la Fuente); Alicia Edith Hermosillo de la Torre, Kalina Isela Martínez Martínez (Universidad Autónoma de Aguascalientes); Rebeca Guzmán Saldaña (Universidad Autónoma del Estado de Hidalgo); Ana María Martínez Jérez (Universidad Autónoma de Tamaulipas); Adrián Abrego Ramírez (Universidad Cuauhtémoc y Universidad Politécnica de Aguascalientes); Ma. Socorro Durán, Gustavo Pérez Tarango, María Alicia Zavala Berbena (Universidad De La Salle Bajío, campus Campestre); Rogaciano González González, Raúl A. Gutiérrez-García, Maria Abigail Paz Pérez (Universidad De La Salle Bajío, campus Salamanca); Anabell Covarrubias Díaz-Couder (Universidad La Salle Noroeste); Sinead Martínez Ruiz (Universidad La Salle Pachuca); Netherlands: Pim Cuijpers (PI), Eirini Karyotaki (VU University Amsterdam); Northern Ireland: Siobhan O’Neill (PI) (School of Psychology, Ulster University); Tony Bjourson, Coral Lapsley, Margaret McLafferty, Elaine Murray (School of Biomedical Sciences, Ulster University); South Africa: Dan J. Stein (PI), (Department of Psychiatry and Mental Health, MRC Unit on Risk & Resilience in Mental Disorders, University of Cape Town); Christine Lochner, Janine Roos (MRC Unit on Risk and Resilience in Mental Disorders, Department of Psychiatry, Stellenbosch University); Jason Bantjes, Elsie Breet (Department of Psychology, Stellenbosch University); Spain: The UNIVERSAL study Group (Universidad y Salud Mental) includes: Jordi Alonso (PI), Gemma Vilagut, Philippe Mortier (IMIM-Hospital del Mar Medical Research Institute/CIBERESP); Itxaso Alayo, Laura Ballester, Gabriela Barbaglia Maria Jesús Blasco, Pere Castellví, Ana Isabel Cebrià, Carlos García-Forero, Andrea Miranda-Mendizábal, Oleguer Parès-Badell (Pompeu Fabra University); José Almenara, Carolina Lagares (Cadiz University), Enrique Echeburúa, Andrea Gabilondo, Álvaro Iruin (Basque Country University); María Teresa Pérez-Vázquez, José Antonio Piqueras, Victoria Soto-Sanz, Jesús Rodríguez-Marín (Miguel Hernández University); and Miquel Roca, Margarida Gili, Margarida Vives (Illes Balears University); United States: Randy P. Auerbach (PI), Claude Mellins (Columbia University); Ronald C. Kessler (PI), (Harvard Medical School); Jennifer G. Green (Boston University); Matthew K. Nock (Harvard University); Stephanie Pinder-Amaker (McLean Hospital and Harvard Medical School); Alan M. Zaslavsky (Harvard Medical School). A complete list of all WMH-ICS publications can be found at: http://www.hcp.med.harvard.edu/wmh/college_student_survey.php.

## Data Availability Statement

The data analyzed in this study is subject to the following licenses/restrictions: The WMH-ICS data sharing agreement limits access of this data to members of the consortium. Requests to access these datasets should be directed to RK, kessler@hcp.med.harvard.edu.

## Ethics Statement

The studies involving human participants were reviewed and approved by Human Research Ethics Committee, Curtin University, Australia Commissie Medische Ethiek-KU Leuven, Katholieke Universiteit (KU) Leuven, Belgium Ethik-Kommission der FAU, Friedrich-Alexander Universitat (FAU) Erlangen-Nurnberg, Germany Survey and Behavioural Research Ethics Committee, Chinese University of Hong Kong, Hong Kong Comité de Ética e Investigación, Instituto Nacional de Psiquiatría Ramón de la Fuente, Mexico Research Ethics Committee, Ulster University, Northern Ireland UCT-Human Research Ethics Committee UCT; Stellenbosch University-Health Research Ethics Committee, University of Cape Town (UCT), South Africa Parc de Salut MAR – Comité Ético de Investigación Clínica, Institut Hospital del Mar d’Investigacions Mèdiques (IMIM), Spain Partners Human Research Committee/Institutional Review Board, Partners HealthCare, McLean Hospital, Harvard Medical School, United States. Written informed consent from the participants’ legal guardian/next of kin was not required to participate in this study in accordance with the national legislation and the institutional requirements.

## Author Contributions

The named co-authors fulfilled the requirements of authorship either by providing data or working on data analysis. EK, NS, and RK produced the first draft and the final revision reflects key comments on the intellectual content from all co-authors. All authors contributed to the article and approved the submitted version. RK accepted the responsibility of taking any steps needed to verify the accuracy and integrity of any part of this work.

## Conflict of Interest

In the past 3 years, RK received support for his epidemiological studies from Sanofi Aventis; was a consultant for Datastat, Inc, Sage Pharmaceuticals, and Takeda. DE reports to have received consultancy fees or served on the scientific advisory board of several companies such as Minddistrict, Sanofi, Lantern, Schön Kliniken, German health insurance companies (BARMER and Techniker Krankenkasse), and chambers of psychotherapists. DE is a stakeholder of the “Institute for Online Health Trainings,” a company aiming to transfer scientific knowledge related to digital mental health to be applied in routine health care. DS has received research grants and/or honoraria from Lundbeck and Sun.

The remaining authors declare that the research was conducted in the absence of any commercial or financial relationships that could be construed as a potential conflict of interest.
