# Supplementary material for: Sources of Stress and Their Associations With Mental Disorders Among College Students: Results of the World Health Organization World Mental Health Surveys International College Student Initiative
Source: Front Psychol. 2020 Jul 30;11:1759. doi: 10.3389/fpsyg.2020.01759 (PMC7406671; doi:10.3389/fpsyg.2020.01759)
Supplement: Supplementary file 1 [file Data_Sheet_1.docx]

| **Supplementary Table 1. The WMH-ICS surveys included in the analysis** | | | | | | | | |
| --- | --- | --- | --- | --- | --- | --- | --- | --- |
|  |  |  |  |  |  |  |  |  |
| **Country** | **Number of participating universities** | **Total size of the universities** | **Number of first-year students eligible** | **Number of first-year student respondents^1^** | **Response Rate^2^** | **Number of first-year students in analyses^3^** | **Survey Field Dates** | **Sampling and procedures** |
| **Australia** | 1 public | ~ 45,000 | 13,752 | 1,202 | 8.7% | 1,048 | 2016-17 | All first-year students were invited to participate through e-mail. Five reminder emails were sent with personalized links to the survey. Conditional incentives were applied (movie passes). |
| **Belgium** | 1 public | ~ 45,000 | 13,103 | 5,844 | 44.6% | 5,703 | 2014-17 | All first-year students between 2014-2016 were invited for a psycho-medical check-up in the student mental health center. Surveys were completed in the waiting room. Students who did not show up for the psycho-medical check-up received up to eight reminder emails. Conditional incentives were applied (store credit coupons). In 2016-2017, all first-year students were invited to participate through e-mail (up to eight emails). |
| **Germany** | 2 public | ~ 40,000 | 11,210 | 1,757 | 15.7% | 1,707 | 2016-18 | All first-year students were invited to participate through e-mail. Six reminder emails were sent with personalized links to the survey. Conditional incentives were applied (store credit coupons). |
| **Hong Kong** | 1 public | ~ 3,000 | 519 | 208 | 40.1% | 208 | 2017 | All first-year full-time students were invited to participate through email and campus posters with QR codes for electronically registering for participation by providing university email address. After verification by university research team of the email addresses being provided, individual survey links were sent to the students. Up to five reminder emails, each one week apart, were sent for non-responding or incomplete surveys. All respondents who completed surveys were given HKD100 (=USD 12.8) shopping coupons as incentives. |
| **Mexico** | 5 private/4 public | ~ 28,000 | 10,747 | 8,076 | 75.1% | 8,053 | 2016-18 | All first-year students were eligible for the survey. Initial contact differed by university: survey included in an obligatory health evaluation (2 universities), as part of obligatory group tutoring sessions (1 university), or as part of required classes (3 universities) or teacher evaluations (2 universities) and direct invitations from university administrators (1 university). Two universities sent reminder emails (tutors sent out emails to their tutees; in a required class of personal development, reminders were sent out by faculty). No incentives were applied for baseline surveys. |
| **Northern Ireland** | 1 public | ~ 25,000 | 4,359 | 739 | 17.0% | 711 | 2015 | All first-year students due to register were invited to participate. Following registration, ID numbers and links to the survey were provided. Five reminder emails/text messages were sent with personalized links to the survey. A 6^th^ reminder involved a researcher telephoning non-responders. All responders were entered into a number of draws to win an iPad. |

| **Supplementary Table 1 continued. The WMH-ICS surveys included in the analysis** | | | | | | | | |
| --- | --- | --- | --- | --- | --- | --- | --- | --- |
|  |  |  |  |  |  |  |  |  |
| **Country** | **Number of participating universities** | **Total size of the universities** | **Number of first-year students eligible** | **Number of first-year student respondents^1^** | **Response Rate^2^** | **Number of first-year students in analyses^3^** | **Survey Field Dates** | **Sampling and procedures** |
| **South Africa** | 1 public | ~ 30,000 | 5,338 | 686 | 12.9% | 666 | 2015 | All first-year students were invited to participate through e-mail. Eight reminder emails and one text message were sent with personalized links to the survey. Conditional incentives were applied (5x R1000 draw). |
| **Spain** | 5 public | ~ 96,000 | 16,332 | 2,118 | 13.0% | 2,046 | 2014-15 | All first-year students 18-24 were eligible for the survey. Initial contact differed by university (information stands, information sessions in classrooms, through the university's website, personalized email from university representatives). Four reminder emails were sent with personalized links to the survey. Conditional monetary incentives were applied. Additionally, an end-game strategy was implemented by selecting a random proportion of non-respondents and offering all of them a monetary incentive. |
| **United States** | 3 private | ~ 21,800 | 4,382 | 739 | 16.9% | 700 | 2015-16 | All first-year students were invited to participate through e-mail. Three reminder emails were sent with personalized links to the survey. Conditional incentives were applied (gift cards). |
| **Total** | 8 private/ 16 public | ~ 333,800 | 79,742 | 21,369 | 45.6% | 20,842 | 2014-18 |  |
|  |  |  |  |  |  |  |  |  |

Abbreviations. WMH-ICS, World Mental Health College Student Initiative.

^1^Full respondents.

^2^Weighted by achieved sample size.

^3^Analysis was restricted to full-time students reporting male or female gender.

| **Supplementary Table 2. Sample sizes in each country** | | | | | |
| --- | --- | --- | --- | --- | --- |
|  |  |  |  |  |  |
|  | **Number of colleges** |  | **%** |  | **(n)** |
| **Country** |  |  |  |  |  |
| Australia | 1 |  | 5.0 |  | (1,048) |
| Belgium | 1 |  | 27.4 |  | (5,703) |
| Germany | 2 |  | 8.2 |  | (1,707) |
| Hong Kong | 1 |  | 1.0 |  | (208) |
| Mexico | 9 |  | 38.6 |  | (8,053) |
| Northern Ireland | 1 |  | 3.4 |  | (711) |
| South Africa | 1 |  | 3.2 |  | (666) |
| Spain | 5 |  | 9.8 |  | (2,046) |
| USA | 3 |  | 3.4 |  | (700) |
| **Total** | 24 |  | 100.0 |  | (20,842) |
|  |  |  |  |  |  |

| **Supplementary Table 3. Sample characteristics (n = 20,842)** | | | | |
| --- | --- | --- | --- | --- |
|  | | | | |
|  | **%** | **(SE)** |  | **(n)^1^** |
| **Gender** |  |  |  |  |
| Female | 54.7 | (0.4) |  | (12,561) |
| **Age** |  |  |  |  |
| 16-18 y | 57.6 | (0.4) |  | (12312) |
| 19 y | 24.6 | (0.3) |  | (4944) |
| 20 y or more | 17.8 | (0.3) |  | (3586) |
| **Parental education** |  |  |  |  |
| High | 55.9 | (0.5) |  | (11605) |
| Medium | 25.1 | (0.4) |  | (5235) |
| Low | 19.0 | (0.4) |  | (4001) |
| **Parents not married or parent(s) deceased** | 24.6 | (0.4) |  | (5222) |
| **Religion** |  |  |  |  |
| Christian | 70.6 | (0.4) |  | (14685) |
| No religion | 25.2 | (0.4) |  | (5288) |
| Another religion | 4.2 | (0.2) |  | (869) |
| **Sexual orientation** |  |  |  |  |
| Heterosexual – no same-sex attraction | 76.2 | (0.4) |  | (15687) |
| Heterosexual – some same-sex attraction | 12.7 | (0.3) |  | (2839) |
| Non-heterosexual - no same-sex intercourse | 6.9 | (0.2) |  | (1447) |
| Non-heterosexual with same-sex intercourse | 4.2 | (0.2) |  | (868) |
| **Self-reported ranking in high school** |  |  |  |  |
| Top 50% | 94.0 | (0.2) |  | (1612) |
| Bottom 50% | 6.0 | (0.2) |  | (1230) |
|  |  |  |  |  |

Note. Sample proportions are based on weighted data. See the text for a description of weighting. Abbreviations. SE, MI-adjusted standard error.

^1^Sample sizes are unweighted.

| **Supplementary Table 4. Univariable associations of perceived stress with 12-month prevalence estimates of DSM-IV major depressive disorder in the WMH-ICS sample (n=20,842)^1^** | | | | | | | | |
| --- | --- | --- | --- | --- | --- | --- | --- | --- |
|  | | | | | | | | |
|  | **Model 1** | |  | **Model 2** | |  | **Model 3** | |
|  | **OR** | **(95% CI)** |  | **OR** | **(95% CI)** |  | **OR** | **(95% CI)** |
| **I. Financial situation** |  |  |  |  |  |  |  |  |
| Any |  |  |  | 0.9 | (0.8-1.1) |  |  |  |
| Linear | 1.4* | (1.3-1.5) |  | 1.4* | (1.4-1.5) |  |  |  |
| Low spline |  |  |  |  |  |  |  |  |
| **II. Own health** |  |  |  |  |  |  |  |  |
| Any |  |  |  | 1.1 | (1.0-1.3) |  |  |  |
| Linear | 1.4* | (1.4-1.5) |  | 1.4* | (1.3-1.5) |  |  |  |
| Low spline |  |  |  |  |  |  |  |  |
| **III. Love life** |  |  |  |  |  |  |  |  |
| Any |  |  |  | 0.7* | (0.6-0.8) |  | 1.2* | (1.0-1.3) |
| Linear | 1.6* | (1.5-1.7) |  | 1.7* | (1.6-1.8) |  |  |  |
| Low spline |  |  |  |  |  |  | 1.7* | (1.6-1.8) |
| **IV. Relationships with family** |  |  |  |  |  |  |  |  |
| Any |  |  |  | 1.2* | (1.0-1.4) |  | 1.7* | (1.5-1.9) |
| Linear | 1.5* | (1.4-1.6) |  | 1.4* | (1.4-1.5) |  |  |  |
| Low spline |  |  |  |  |  |  | 1.4* | (1.4-1.5) |
| **V. Relationships at school or work** |  |  |  |  |  |  |  |  |
| Any |  |  |  | 1.0 | (0.8-1.1) |  |  |  |
| Linear | 1.5* | (1.5-1.6) |  | 1.6* | (1.5-1.7) |  |  |  |
| Low spline |  |  |  |  |  |  |  |  |
| **VI. Problems of loved ones** |  |  |  |  |  |  |  |  |
| Any |  |  |  | 0.8* | (0.7-0.9) |  | 1.1 | (1.0-1.3) |
| Linear | 1.4* | (1.3-1.4) |  | 1.4* | (1.3-1.5) |  |  |  |
| Low spline |  |  |  |  |  |  | 1.4* | (1.3-1.5) |
|  |  |  |  |  |  |  |  |  |

Abbreviations. WMH-ICS, World Mental Health College Student Initiative; OR, odds ratio; CI, confidence interval.

*Significant at the .05-level, using MI-adjusted tests.

^1^All models based on weighted data controlling for the socio-demographic variables in Supplementary Table 3. Any = 0-1 dichotomy coded 0 for respondents who reported no stress in the life area and coded 1 for respondents who reported mild, moderate, severe, or very severe stress in the life area; Linear = 0-4 variable for stress severity reported by respondent: no stress (0), mild (1), moderate (2), severe (3), or very severe (4) stress in the life area; Low spline = 1-4 variable for respondents who reported no stress (1), mild (1), moderate (2), severe (3), or very severe (4) stress in the life area.

| **Supplementary Table 5. Univariable associations of perceived stress with 12-month prevalence estimates of DSM-IV generalized anxiety disorder in the WMH-ICS sample (n=20,842)^1^** | | | | | | | | |
| --- | --- | --- | --- | --- | --- | --- | --- | --- |
|  | | | | | | | | |
|  | **Model 1** | |  | **Model 2** | |  | **Model 3** | |
|  | **OR** | **(95% CI)** |  | **OR** | **(95% CI)** |  | **OR** | **(95% CI)** |
| **I. Financial situation** |  |  |  |  |  |  |  |  |
| Any |  |  |  | 0.7* | (0.6-0.8) |  | 1.2* | (1.0-1.3) |
| Linear | 1.5* | (1.5-1.6) |  | 1.7* | (1.6-1.8) |  |  |  |
| Low spline |  |  |  |  |  |  | 1.7* | (1.6-1.8) |
| **II. Own health** |  |  |  |  |  |  |  |  |
| Any |  |  |  | 1.7* | (1.6-1.8) |  |  |  |
| Linear | 1.7* | (1.6-1.7) |  | 1.0 | (0.8-1.2) |  |  |  |
| Low spline |  |  |  |  |  |  |  |  |
| **III. Love life** |  |  |  |  |  |  |  |  |
| Any |  |  |  | 0.7* | (0.6-0.8) |  | 1.2* | (1.1-1.4) |
| Linear | 1.6* | (1.5-1.7) |  | 1.7* | (1.6-1.8) |  |  |  |
| Low spline |  |  |  |  |  |  | 1.7* | (1.6-1.8) |
| **IV. Relationships with family** |  |  |  |  |  |  |  |  |
| Any |  |  |  | 1.1 | (0.9-1.3) |  |  |  |
| Linear | 1.6* | (1.5-1.7) |  | 1.6* | (1.5-1.7) |  |  |  |
| Low spline |  |  |  |  |  |  |  |  |
| **V. Relationships at school or work** |  |  |  |  |  |  |  |  |
| Any |  |  |  | 1.0 | (0.9-1.2) |  |  |  |
| Linear | 1.7* | (1.6-1.8) |  | 1.7* | (1.6-1.8) |  |  |  |
| Low spline |  |  |  |  |  |  |  |  |
| **VI. Problems of loved ones** |  |  |  |  |  |  |  |  |
| Any |  |  |  | 0.7* | (0.6-0.8) |  | 1.1 | (1.0-1.3) |
| Linear | 1.6* | (1.5-1.6) |  | 1.7* | (1.6-1.8) |  |  |  |
| Low spline |  |  |  |  |  |  | 1.7* | (1.6-1.8) |
|  |  |  |  |  |  |  |  |  |

Abbreviations. WMH-ICS, World Mental Health College Student Initiative; OR, odds ratio; CI, confidence interval.

*Significant at the .05-level, using MI-adjusted tests.

^1^All models based on weighted data controlling for the socio-demographic variables in Supplementary Table 3. Any = 0-1 dichotomy coded 0 for respondents who reported no stress in the life area and coded 1 for respondents who reported mild, moderate, severe, or very severe stress in the life area; Linear = 0-4 variable for respondents who reported no stress (0), mild (1), moderate (2), severe (3), or very severe (4) stress in the life area; Low spline = 1-4 variable for respondents who reported no stress (1), mild (1), moderate (2), severe (3), or very severe (4) stress in the life area.

| **Supplementary Table 6. Univariable associations of perceived stress with 12-month prevalence estimates of DSM-IV bipolar disorder in the WMH-ICS sample (n=20,842)^1^** | | | | | | | | |
| --- | --- | --- | --- | --- | --- | --- | --- | --- |
|  | | | | | | | | |
|  | **Model 1** | |  | **Model 2** | |  | **Model 3** | |
|  | **OR** | **(95% CI)** |  | **OR** | **(95% CI)** |  | **OR** | **(95% CI)** |
| **I. Financial situation** |  |  |  |  |  |  |  |  |
| Any |  |  |  | 0.9 | (0.6-1.2) |  |  |  |
| Linear | 1.7* | (1.6-1.8) |  | 1.7* | (1.6-1.9) |  |  |  |
| Low spline |  |  |  |  |  |  |  |  |
| **II. Own health** |  |  |  |  |  |  |  |  |
| Any |  |  |  | 1.4* | (1.0-1.9) |  | 2.1* | (1.6-2.6) |
| Linear | 1.6* | (1.5-1.7) |  | 1.5* | (1.3-1.6) |  |  |  |
| Low spline |  |  |  |  |  |  | 1.5* | (1.3-1.6) |
| **III. Love life** |  |  |  |  |  |  |  |  |
| Any |  |  |  | 0.7 | (0.5-1.0) |  |  |  |
| Linear | 1.6* | (1.5-1.8) |  | 1.7* | (1.6-1.9) |  |  |  |
| Low spline |  |  |  |  |  |  |  |  |
| **IV. Relationships with family** |  |  |  |  |  |  |  |  |
| Any |  |  |  | 1.4* | (1.1-1.9) |  | 2.3* | (1.8-3.0) |
| Linear | 1.7* | (1.6-1.8) |  | 1.6* | (1.5-1.8) |  |  |  |
| Low spline |  |  |  |  |  |  | 1.6* | (1.5-1.8) |
| **V. Relationships at school or work** |  |  |  |  |  |  |  |  |
| Any |  |  |  | 1.2 | (0.9-1.6) |  |  |  |
| Linear | 1.7* | (1.6-1.8) |  | 1.6* | (1.4-1.8) |  |  |  |
| Low spline |  |  |  |  |  |  |  |  |
| **VI. Problems of loved ones** |  |  |  |  |  |  |  |  |
| Any |  |  |  | 1.0 | (0.8-1.5) |  |  |  |
| Linear | 1.5* | (1.4-1.6) |  | 1.5* | (1.4-1.6) |  |  |  |
| Low spline |  |  |  |  |  |  |  |  |
|  |  |  |  |  |  |  |  |  |

Abbreviations. WMH-ICS, World Mental Health College Student Initiative; OR, odds ratio; CI, confidence interval.

*Significant at the .05-level, using MI-adjusted tests.

^1^All models based on weighted data controlling for the socio-demographic variables in Supplementary Table 3. Any = 0-1 dichotomy coded 0 for respondents who reported no stress in the life area and coded 1 for respondents who reported mild, moderate, severe, or very severe stress in the life area; Linear = 0-4 variable for respondents who reported no stress (0), mild (1), moderate (2), severe (3), or very severe (4) stress in the life area; Low spline = 1-4 variable for respondents who reported no stress (1), mild (1), moderate (2), severe (3), or very severe (4) stress in the life area.

| **Supplementary Table 7. Univariable associations of perceived stress with 12-month prevalence estimates of DSM-IV panic disorder in the WMH-ICS sample (n=20,842)^1^** | | | | | | | | |
| --- | --- | --- | --- | --- | --- | --- | --- | --- |
|  | | | | | | | | |
|  | **Model 1** | |  | **Model 2** | |  | **Model 3** | |
|  | **OR** | **(95% CI)** |  | **OR** | **(95% CI)** |  | **OR** | **(95% CI)** |
| **I. Financial situation** |  |  |  |  |  |  |  |  |
| Any |  |  |  | 0.8 | (0.6-1.1) |  |  |  |
| Linear | 1.5* | (1.4-1.6) |  | 1.6* | (1.4-1.7) |  |  |  |
| Low spline |  |  |  |  |  |  |  |  |
| **II. Own health** |  |  |  |  |  |  |  |  |
| Any |  |  |  | 1.1 | (0.8-1.6) |  |  |  |
| Linear | 1.7* | (1.6-1.8) |  | 1.7* | (1.5-1.8) |  |  |  |
| Low spline |  |  |  |  |  |  |  |  |
| **III. Love life** |  |  |  |  |  |  |  |  |
| Any |  |  |  | 0.6* | (0.5-0.9) |  | 1.0 | (0.8-1.2) |
| Linear | 1.3* | (1.2-1.4) |  | 1.5* | (1.3-1.7) |  |  |  |
| Low spline |  |  |  |  |  |  | 1.5* | (1.3-1.7) |
| **IV. Relationships with family** |  |  |  |  |  |  |  |  |
| Any |  |  |  | 1.1 | (0.8-1.5) |  |  |  |
| Linear | 1.4* | (1.3-1.5) |  | 1.4* | (1.2-1.5) |  |  |  |
| Low spline |  |  |  |  |  |  |  |  |
| **V. Relationships at school or work** |  |  |  |  |  |  |  |  |
| Any |  |  |  | 1.0 | (0.7-1.3) |  |  |  |
| Linear | 1.4* | (1.3-1.5) |  | 1.4* | (1.2-1.6) |  |  |  |
| Low spline |  |  |  |  |  |  |  |  |
| **VI. Problems of loved ones** |  |  |  |  |  |  |  |  |
| Any |  |  |  | 0.7* | (0.5-1.0) |  | 1.1 | (0.8-1.4) |
| Linear | 1.4* | (1.3-1.5) |  | 1.5* | (1.3-1.6) |  |  |  |
| Low spline |  |  |  |  |  |  | 1.5* | (1.3-1.6) |
|  |  |  |  |  |  |  |  |  |

Abbreviations. WMH-ICS, World Mental Health College Student Initiative; OR, odds ratio; CI, confidence interval.

*Significant at the .05-level, using MI-adjusted tests.

^1^All models based on weighted data controlling for the socio-demographic variables in Supplementary Table 3. Any = 0-1 dichotomy coded 0 for respondents who reported no stress in the life area and coded 1 for respondents who reported mild, moderate, severe, or very severe stress in the life area; Linear = 0-4 variable for respondents who reported no stress (0), mild (1), moderate (2), severe (3), or very severe (4) stress in the life area; Low spline = 1-4 variable for respondents who reported no stress (1), mild (1), moderate (2), severe (3), or very severe (4) stress in the life area.

| **Supplementary Table 8. Univariable associations of perceived stress with 12-month prevalence estimates of DSM-IV alcohol use disorder in the WMH-ICS sample (n=20,842)^1^** | | | | | | | | |
| --- | --- | --- | --- | --- | --- | --- | --- | --- |
|  | | | | | | | | |
|  | **Model 1** | |  | **Model 2** | |  | **Model 3** | |
|  | **OR** | **(95% CI)** |  | **OR** | **(95% CI)** |  | **OR** | **(95% CI)** |
| **I. Financial situation** |  |  |  |  |  |  |  |  |
| Any |  |  |  | 0.9 | (0.7-1.2) |  |  |  |
| Linear | 1.3* | (1.2-1.4) |  | 1.4* | (1.2-1.5) |  |  |  |
| Low spline |  |  |  |  |  |  |  |  |
| **II. Own health** |  |  |  |  |  |  |  |  |
| Any |  |  |  | 0.9 | (0.7-1.2) |  |  |  |
| Linear | 1.3* | (1.2-1.3) |  | 1.3* | (1.2-1.4) |  |  |  |
| Low spline |  |  |  |  |  |  |  |  |
| **III. Love life** |  |  |  |  |  |  |  |  |
| Any |  |  |  | 1.0 | (0.8-1.3) |  |  |  |
| Linear | 1.4* | (1.3-1.5) |  | 1.4* | (1.3-1.5) |  |  |  |
| Low spline |  |  |  |  |  |  |  |  |
| **IV. Relationships with family** |  |  |  |  |  |  |  |  |
| Any |  |  |  | 0.9 | (0.7-1.1) |  |  |  |
| Linear | 1.3* | (1.2-1.4) |  | 1.3* | (1.2-1.4) |  |  |  |
| Low spline |  |  |  |  |  |  |  |  |
| **V. Relationships at school or work** |  |  |  |  |  |  |  |  |
| Any |  |  |  | 1.2 | (0.9-1.5) |  |  |  |
| Linear | 1.3* | (1.2-1.4) |  | 1.2* | (1.1-1.4) |  |  |  |
| Low spline |  |  |  |  |  |  |  |  |
| **VI. Problems of loved ones** |  |  |  |  |  |  |  |  |
| Any |  |  |  | 1.1 | (0.8-1.4) |  |  |  |
| Linear | 1.3* | (1.2-1.3) |  | 1.2* | (1.1-1.3) |  |  |  |
| Low spline |  |  |  |  |  |  |  |  |
|  |  |  |  |  |  |  |  |  |

Abbreviations. WMH-ICS, World Mental Health College Student Initiative; OR, odds ratio; CI, confidence interval.

*Significant at the .05-level, using MI-adjusted tests.

^1^All models based on weighted data controlling for the socio-demographic variables in Supplementary Table 3. Any = 0-1 dichotomy coded 0 for respondents who reported no stress in the life area and coded 1 for respondents who reported mild, moderate, severe, or very severe stress in the life area; Linear = 0-4 variable for respondents who reported no stress (0), mild (1), moderate (2), severe (3), or very severe (4) stress in the life area; Low spline = 1-4 variable for respondents who reported no stress (1), mild (1), moderate (2), severe (3), or very severe (4) stress in the life area.

| **Supplementary Table 9. Univariable associations of perceived stress with 12-month prevalence estimates of DSM-IV drug use disorder in the WMH-ICS sample (n=20,842)^1^** | | | | | | | | |
| --- | --- | --- | --- | --- | --- | --- | --- | --- |
|  | | | | | | | | |
|  | **Model 1** | |  | **Model 2** | |  | **Model 3** | |
|  | **OR** | **(95% CI)** |  | **OR** | **(95% CI)** |  | **OR** | **(95% CI)** |
| **I. Financial situation** |  |  |  |  |  |  |  |  |
| Any |  |  |  | 0.9 | (0.6-1.4) |  |  |  |
| Linear | 1.5* | (1.3-1.6) |  | 1.5* | (1.3-1.7) |  |  |  |
| Low spline |  |  |  |  |  |  |  |  |
| **II. Own health** |  |  |  |  |  |  |  |  |
| Any |  |  |  | 1.2 | (0.8-1.8) |  |  |  |
| Linear | 1.3* | (1.2-1.5) |  | 1.3* | (1.1-1.5) |  |  |  |
| Low spline |  |  |  |  |  |  |  |  |
| **III. Love life** |  |  |  |  |  |  |  |  |
| Any |  |  |  | 0.5 | (0.4-0.8) |  | 0.9 | (0.6-1.3) |
| Linear | 1.4* | (1.3-1.6) |  | 1.7* | (1.5-1.9) |  |  |  |
| Low spline |  |  |  |  |  |  | 1.7* | (1.5-1.9) |
| **IV. Relationships with family** |  |  |  |  |  |  |  |  |
| Any |  |  |  | 1.2 | (0.8-1.9) |  |  |  |
| Linear | 1.5* | (1.3-1.6) |  | 1.4* | (1.2-1.6) |  |  |  |
| Low spline |  |  |  |  |  |  |  |  |
| **V. Relationships at school or work** |  |  |  |  |  |  |  |  |
| Any |  |  |  | 0.9 | (0.6-1.4) |  |  |  |
| Linear | 1.4* | (1.2-1.5) |  | 1.4* | (1.2-1.6) |  |  |  |
| Low spline |  |  |  |  |  |  |  |  |
| **VI. Problems of loved ones** |  |  |  |  |  |  |  |  |
| Any |  |  |  | 1.4 | (0.9-2.2) |  |  |  |
| Linear | 1.3* | (1.2-1.4) |  | 1.2* | (1.1-1.4) |  |  |  |
| Low spline |  |  |  |  |  |  |  |  |
|  |  |  |  |  |  |  |  |  |

Abbreviations. WMH-ICS, World Mental Health College Student Initiative; OR, odds ratio; CI, confidence interval.

*Significant at the .05-level, using MI-adjusted tests.

^1^All models based on weighted data controlling for the socio-demographic variables in Supplementary Table 3. Any = 0-1 dichotomy coded 0 for respondents who reported no stress in the life area and coded 1 for respondents who reported mild, moderate, severe, or very severe stress in the life area; Linear = 0-4 variable for respondents who reported no stress (0), mild (1), moderate (2), severe (3), or very severe (4) stress in the life area; Low spline = 1-4 variable for respondents who reported no stress (1), mild (1), moderate (2), severe (3), or very severe (4) stress in the life area.
